# Timelines of the “free-particle” and “fixed-particle” models of stone-formation: theoretical and experimental investigations

**DOI:** 10.1007/s00240-016-0946-x

**Published:** 2016-12-03

**Authors:** D. J. Kok, W. Boellaard, Y. Ridwan, V. A. Levchenko

**Affiliations:** 1 Department of Urology, Erasmus MC, Rotterdam, The Netherlands; 2 Department of Radiology, Erasmus MC, Rotterdam, The Netherlands; 3 Centre for Accelerator Science, Australian Nuclear Science and Technology Organisation, Kirrawee, Australia

**Keywords:** Urolithiasis, Age, Isotope, Calcium oxalate, Calcium phosphate, Stone formation

## Abstract

Two major theories on renal stone formation will be reviewed, the “free-particle” and “fixed-particle” mechanisms. These theories combine data on intrinsic factors (inborn metabolic errors), extrinsic factors (diet), renal cell responses and the physico-chemistry and biochemistry of urine into mechanisms of stone formation. This paper describes the specific role of time in both mechanisms. The timeline of crystal- and stone formation was deducted from literature data and was measured for two stones using radioisotope decay analysis. The stones of similar size and composition showed, respectively, a timeline of a few years and a development that took decades. In combination with data on stone architecture and patient characteristics these timelines are explained using the free-particle and fixed-particle mechanisms. Consideration of the timeline of stone formation has clinical implications. We conclude that the fixed-particle mechanism can be a slow process where decades pass between the first formation of a precipitate in the renal interstitium and the clinical presentation of the stone. Added to the fact that the mechanism of this initial precipitation is still ill defined, the conditions that started fixed-particle stone formation in an individual patient can be obscure. Blood and urine analysis in such patients does not necessarily reveal the individual’s risk for recurrence as lifestyle may have changed over time. This is in fact what defines the so-called idiopathic stoneformers. For these patients, prevention of outgrowth of previously formed precipitates, papillary plaques, may be more relevant than prevention of new plaque formation. In contrast, a patient who has formed a stone in a relatively short time through the free-particle mechanism is more likely to show abnormal values in blood and urine that explain the starting event of stone formation. In these patients, measurement of such values provides useful information to guide preventive measures.

## Introduction

Why does someone form a stone? A basic requirement for stone formation to start and proceed in the urinary tract is that at some point and time the amount of stone mineral that is present in solution exceeds the amount that can be sustained. Thermodynamics dictate that such a situation is not stable and that, after some time, the instability is resolved by forming a precipitate. This event may occur inside renal tubules or inside the renal interstitium, the latter specifically in papillary tips around the bends in the longest loops of Henle. The two locations differ with respect to the local fluid dynamics. The time that is available to form a precipitate in the fluid that is passing through a nephron is limited by the passage time of the fluid through the nephron, which is on a scale of minutes. When a precipitate is not formed within that time frame it will only lead to crystalluria. In addition, when a precipitate is formed inside the nephron its presence must somehow be prolonged, particle retention, otherwise it will still end up as crystalluria. More time is available for precipitation in the static interstitial environment. The beginning particles are not swept along, retention is the norm.

As said, thermodynamics dictate that when the amount of a substance in solution exceeds its solubility, a state of supersaturation, precipitation will occur. The mere fact that a fluid can be supersaturated shows that this precipitation is not instantaneous. There is a threshold for precipitation and the energy contained in the state of supersaturation is needed to surmount this threshold. The rate at which the threshold is taken increases with increasing supersaturation. Inversely, the time that is needed to start precipitation decreases with increasing supersaturation. This means that it will require a higher supersaturation to form a precipitate in urine that is rushing along through a nephron than to form a precipitate in static interstitial fluid where the changes are limited to changes in concentrations and in pH.

In this paper we will look in further detail at this effect of time in stone formation. As framework we will use two theories of stone formation, the free-particle and fixed-particle mechanism [1] with some modification. Free-particle stone formation is defined by us as intratubular precipitation followed by intratubular plug formation and eventually stone formation. The fixed-particle mechanism is defined as the formation of a papillary plaque followed by outgrowth into a stone. This differs from the original free-particle and fixed-particle classification in that fixation of particles inside tubules, for instance to damaged tubular cells, is by us considered to lead to a plug and is classified as a free-particle mechanism.

### Clinical consequences attached to the free-particle and fixed-particle theories

A logical assumption is that it should be possible to prevent stone formation by preventing the initial supersaturation/precipitation. This idea prompted the analysis of risk factors in blood and urine and led to comprehensive models that translate such data into a risk of stone formation [2, 3]. After decades of refinement, some high-risk patients can be identified and helped to prevent recurrence. For patients with hereditary hyperoxaluria or patients with primary hyperparathyroidism the risk factors are clear. For hereditary hyperoxaluria, correction of the risk factor unfortunately is a problem, but primary hyperparathyroidism can be treated and this does normalize the risk factors (hypercalcemia and hypercalciuria) and does prevent new stone formation. For most stoneformers, however, there is a large overlap in risk factor values with people who do not form stones, and urine supersaturation often is not an obvious cause for stone formation [4, 5]. Prime examples are the so-called “idiopathic” stoneformers in risk factor analysis shows a normal situation. This holds true both for the traditional supersaturation-linked risk factors in blood and urine and for the more elaborate risk models that include effects of urine on crystallization kinetics. There exist clear-cut cases, recurrent stoneformers who distinguish themselves by a reduced or even absent capacity to prevent crystal agglomeration, but also patients who have formed only one stone and in whom urine prevents crystal agglomeration just as well as in people who never formed a stone [6–8].

This gray area problem diminishes the eagerness of clinicians to include blood and urine risk analysis in their treatment of stone patients. Together with low patient compliance with therapy, this frustrates the efficacy of preventive measures in general practice [9].

In the whole risk analysis, the individual timeline of the stone formation is usually not regarded. In some patients, the initial step towards stone formation is ongoing and traditional risk factor analysis should have value. In others, the stone formation was initiated a long time ago and the risk for stone growth should be evaluated. To exemplify this role of time, we measured the exact timeline of stone formation using radioisotope decay analysis (see “Materials and methods”).

### Two stone timelines

**Fig. 1:**
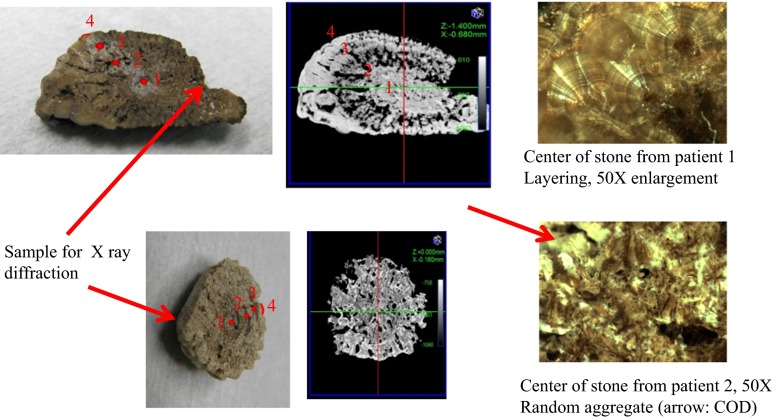
The *top panel* shows the stone taken from patient 1. On the *left side* a photo of the stone after it was cut into two halves. In the *middle* is a CT photo taken of the middle plane of the intact stone. On the *right hand* is a micrograph of the center of the stone. Guided by the CT scan, the points* 1*–*4* were chosen to take samples for age determination. Site 1 is inside the mineral center of the stone. Site 2 is where the mineral center goes over into a more organic layer. Site 3 is at the other side of that organic layer. Site 4 is in the mineral outer layer of the stone. Along the same axis PIXE analysis was performed to determine the relative presence of elements. The *lower panel* shows the stone taken from patient 2. The CT does not show the layering as in stone 1. At four sites along the axis from the stone center to the outer surface samples were taken for the age determination. PIXE analysis was performed along the same axis

From two patients an intact stone was obtained in November 2013. Both stones had a long axis of approximately 3 cm. Stone characteristics are described in Table 1. A part of the stone, as indicated in Fig. 1, was removed for routine stone analysis by X-ray diffraction. A CT scan was made of each stone at a resolution of 20 µm and a CT video at a resolution of 40 µm. After this the stones were cut in half. For research purposes, stone analysis was then also performed at several internal stone sites using elemental analysis. The X-ray diffraction showed 100% whewellite for stone 1. Elemental analysis along the axis indicated in Fig. 1 showed a low P/Ca ratio at all points. The part removed from stone 2 was a mix of 5% whewellite, 65% weddelite, and 30% apatite. The phosphate/Ca ratio along the axis indicated in Fig. 1 averaged 0.18. Stone 1 had a strongly layered architecture both in the microscopic photo of the center and macroscopically in the CT scan showing alternating layers with high and low mineral content. The CT of stone 2 showed a uniform architecture of mixed mineral/organic material presence throughout the stone. Microscopy revealed a center composed of a random aggregate of crystals and organic material.

**Table 1:** CT, isotope decay-(age) and PIXE (elements)-analysis of the two stones

	Distance (%)	Growth, radius (cm/year)	Growth, volume (mm^3^/year)	P/Ca ratio	Year of deposition (date ± year)	CT opaque (O), translucent (T)	O/T ratio (%/%)
Stone 1							
Site 1	0	–		<0.05	1990.8 ± 0.8	Center O	80/20
Site 2	46	0.090	73	<0.05	1998.5 ± 1.0	Inside border O → T	30/70
Site 3	73	0.100	910		2002.5 ± 1.0	Outside border T → O	30/70
Site 4	100	0.037	890	<0.05	2013.5 ± 0.6	Outside layer O	95/5
Stone 2							
Site 1	0	–		2–3	2005.8 ± 1.1	O with T inclusions	90/10
Site 2	33	0.38	1100	0–0.3	2007.1 ± 1,3	O with T inclusions	90/10
Site 3	66	0.50	4200	0.3–0.6	2008.1 ± 1.2	O with T inclusions	90/10
Site 4	100	0.09	1600	0–0.3	2013.5 ± 0.7	O with T inclusions	90/10

**Fig. 2:**
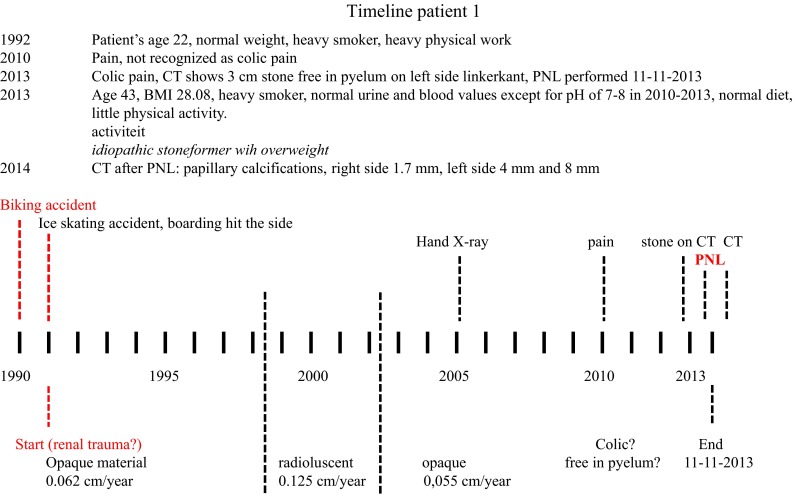
Lifestyle events, clinical data and urinary stone events during two decades in patient 1

The center of stone 1 originated from 1991. That year the patient was 22 years old. He happened to visit our hospital for two consecutive accidents involving a trauma to his flank (Fig. 2). He had a normal weight, was a heavy smoker and performed hard physical labor. From 1991 to 1998 the stone grew at an average rate of 0.09 cm/year as a predominantly mineral layer. Then a distinctly different growth period started, lasting until 2002, during which a predominantly organic layer was formed. While the radial growth rate then was approximately the same (0.1 cm/year) the volume growth (the amount of material deposited) accelerated from ~70 to ~900 mm^3^/year (see Table 1). No clinical data are known for this period. From 2002 until the stone was removed, 2013, a predominantly mineral layer precipitated at approximately the same pace (890 mm^3^/year). Since the stone was bigger, the linear increase was slower than before (0.037 cm/year). In 2010 the patient first noticed pain that with hindsight can be attributed to the stone. Its presence was confirmed in 2013. The metabolic check-up performed in 2013 revealed overweight, little physical activity and normal urine/blood values of stone risk parameters. The conclusion in 2013 was: an idiopathic calcium oxalate stoneformer. After stone removal, the CT showed that three papillary calcifications remained present. As said earlier, the elemental analysis along the 1–4 stone axis showed on average a low P/Ca ratio, of about 0.05 with only a few narrow spikes to 0.2 in the central region of the stone. As the resolution of the element analysis was at best 20 µm, we cannot exclude the presence of microgranules of Ca phosphate in the center. Furthermore we cannot distinguish between P contained in organic materials versus P contained in crystals.

**Fig. 3:**
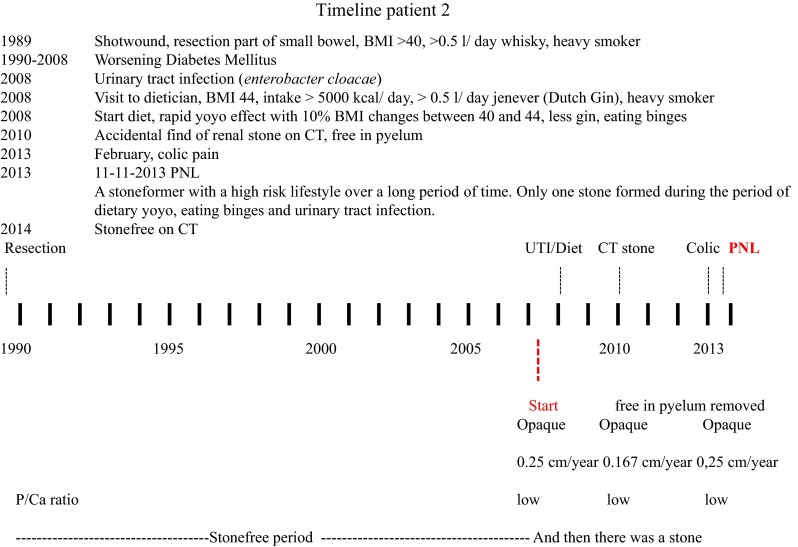
Lifestyle events, clinical data and urinary stone events during two decades in patient 2

The center of stone 2 was from approximately 2006. Compared to stone 1, the stone initially grew at a noticeably higher rate of ~0.38 cm/year (1100 mm^3^/year). At all time-points, the stone increased in size by deposition of both inorganic and organic material at a constant (high) mineral/organic ratio. The patient had a history of obesity and large alcohol intake dating back to 1989 (see Fig. 3). In that year, a partial small bowel resection was performed following a traumatic wound to the stomach area. The patient was followed for development of diabetes during the decades thereafter. His dietary records show a high intake of all food categories (animal protein, fruit, vegetables, carbohydrates, fluids) adding up to a caloric intake of >5000 kcal/day. In 2008 he suffered from a urinary tract infection (*Enterobacter cloacae*). In the stone, the period around 2008 is marked by rapid stone growth both in linear size (0.5 cm/year) and in volume (4200 mm^3^/year). In 2005 he started a weight loss program, reducing the intake of calories and reducing the intake of high alcohol-containing beverages by 0.5 l per day. This program was continued through 2007. It was partial successful but he had setbacks with binge-eating and as a result his BMI yoyo-ed with 10% changes between 40 and 44. The oldest parts of the stone originate from this period (end 2005). In 2010, the stone was discovered on a CT scan lying free in the pyelum. Up until removal, the stone was growing at a reduced rate of 1600 mm^3^/year (0.09 cm/year). In conclusion, a stoneformer with a high-risk lifestyle who did not form a stone during decades of extreme intake of all food categories but did form a stone when the diet pattern became unstable. Periods of dietary restriction, with low renal loads, were followed by periods of binge-eating with extreme renal loads. After stone removal, the kidneys were clear of calcifications. The distribution of elements along the axis constituted by points 1–4 was measured with PIXE. This demonstrated a P/Ca ratio of about 0.3 on average with peaks up to 0.6 (pure apatite). The lowest values were found closer to the surface of the stone.

We will now try to reconcile these data with the theories on stone formation using our classification of the free-particle and fixed-particle mechanisms.

## Models of stone formation

First, a distinct class of stone formation that does not fit into our classification and falls outside the scope of this review is that where the whole stone formation process occurs unrelated to renal tissue, in the urinary tract. Best example is infection stone formation. It does follow the same thermodynamic principles, being driven by a high supersaturation of the urine with respect to struvite and calcium phosphates. This is caused by bacteria that produce urease which increases in, respectively, pH and ammonium concentration [10]. Treatment options are straightforward, remove all crystal material and remove the bacteria. Unfortunately the result is less straightforward as bacteria can hide from antibiotic treatment by developing resistance and by hiding inside urothelial cells [11].

### Free-particle mechanism, tubular plugs

The free-particle mechanism starts with formation of crystals inside the nephron. When this occurs stone formation is not inevitable. Tubular precipitates that are not retained but removed become harmless crystalluria. Retention involves formation of particles that are too large to pass [1, 12] or cellular adhesion to damaged cells [13] possibly caused by oxidative stress [14]. When crystals are retained they can enter the interstitium, as seen both in animal models of stone formation and in patients [15]. They can also become plugs that are found inside the tubule or protruding from the duct of Bellini [16–20]. Such plugs have been found in patients forming a variety of different stone types: calcium phosphates, calcium oxalates, uric acid, cystine, struvite, dihydroxyadenine and even matrix (Table 2). The number of patients with plugs and the number of duct with plugs per patient are highest in distinct patients with primary hyperoxaluria, primary hyperparathyroidism, enteric hyperoxaluria, and distal renal tubular acidosis (dRTA). Except for dRTA, these conditions share the features of an increased serum level and increased renal load of stone-forming compounds like calcium, oxalate or cystine. A high renal load leads to increased intratubular concentrations and a stronger drive to form crystals already early in the passage through the nephron. There is more time for crystallization to start and more time to form large aggregates that may become a plug [1]. Patients with dRTA do not necessarily have increased renal loads but do have a strongly reduced urine concentration of citrate in the distal parts of the nephron. This lowers their ability to prevent formation of large aggregates [6, 7], which enhances the chance of plug formation [1]. Uric acid stoneformers may have increased renal loads of uric acid but most of all they have low urine pH values starting in the distal parts of the nephron that stimulate precipitation of uric acid crystals within the passage time of the tubules.

**Table 2:** Prevalence of plugs and plaques, based on data presented in Linnes [16]

Stone type	Ca phosph	Other	CaOx mal	Struvite	CaOx	UA
*N* patients	12	8	8	9	37	4
Tubular plugs						
>1% plug area	58%	25%	25%	11%	11%	0%
Patients with plugs	75%	50%	50%	33	33	50
Driving abnormality^a^	pH↑	ox↑, cit↓	cit↓	pH↑	Crystal GI↓^b^	
Papillary plaque						
Average surface area %	2.8	2.3	3.1	2.1	3.6	1.7
Driving abnormality^a^					cit↓	

*Other* primary hyperoxaluria, primary hyperparathyroidism. Dihydroxyadenine, unidentifiable crystal, matrix stones; Ca phosph (HyperCalciuria + HyperPhosphaturia + high pH), *CaOx mal* calcium oxalate stoneformers with malabsorption, *UA* uric acid

^a^Driving abnormality obtained from urine analysis

^b^Method used to measure crystal growth did not distinguish between crystal growth and crystal aggregation

Overall, plugs consist of random aggregates that are formed due to an acute high supersaturation with respect to some stone component inside the renal tubules that inherently can be linked to the existing patient’s lifestyle and condition. However, does a plug inevitably lead to the formation of a stone?

Particles that get fixed to the wall inside tubules may be actively removed by macrophages. This involves entry into the interstitium followed by attraction and activation of macrophages [15, 21–23]. This has been observed both in animals and in human patients. The attraction and activation appear to be regulated by organic compounds included in the crystals (the so-called crystal matrix), like osteopontin [24–26]. There are no data that this active removal mechanism may also remove plugs from the duct of Bellini. However, there is indirect evidence that such plugs may be released another way. Papillae often show dilated ducts in the vicinity of ducts that contain a plug [17]. These dilated ducts may have contained plugs that for some reason were removed. Expulsion by urine flow is a possibility. When such expulsion can occur, a major protective effect of the drinking advice might be to remove ductal plugs and timing of the drinking might be important. The existence of such a removal mechanism would explain why patients form many times more plugs than stones.

### Fixed-particle mechanism, papillary plaques

The initial step of papillary plaque formation is thought to be precipitation of calcium phosphate in the interstitium around the bends of the longest loops of Henle [17, 19]. Again compounds like osteopontin are found in plaques [27] but there are no signs of active removal. Through a sequence of alternating accumulations of crystal components and organic material these deposits increase in size following a layered growth pattern. First, this proceeds inside the renal interstitium. It continues when the plaque becomes exposed to the urine space outside the papilla. Since particles in the renal interstitium cannot be voided the finding that drinking enough to produce at least 2 L of urine per day decreases stone formation [28] can in these patients only be related to the stone growth process. What actually starts plaque formation is unclear. The fluid in the interstitium is static. Little is known how its composition changes over time. Possibly there are periods of increased supersaturation following for instance increased reabsorption of calcium and phosphate. It is reasonable to assume that such periods last longer than the nephron passage time. Consequently precipitation requires a lower supersaturation in the interstitium than in the nephron. Precipitation may be stimulated by exposure of an organic surface that acts as a heterogeneous nucleator lowering the precipitation threshold. It has been suggested that this involves stress to renal tissue induced by renal overloading and oxidative damage [27].

Overall, the fixed-particle mechanism contains an initial period of deposition that proceeds outside the dynamics of the urine flow, possibly quite slowly, followed by a growth period when exposure to the urine occurs. In light of the more continuous supply of new material by urine the second part may be faster. The actual growth rate then will depend on the balance of deposition of stone components and deposition of organic material which may stop the crystal growth process temporarily. Stone architecture can be expected to reflect over-time fluctuations, like with the layered aspect of stone 1.

## The timeline of stone formation

Thus, theoretically renal crystal deposition takes minutes, while our data show that stone formation can take decades. Table 3 provides an overview of literature data on observed timelines for precipitation and stone formation. Calcium oxalate crystals are found inside the proximal tubule under extreme conditions like acute and chronic oxalosis both in rat models and in human patients [15, 29]. Formation and retention of these crystals must have occurred within the passage time for that segment, which is less than a minute [1]. Under conditions of less extreme but still high renal loading with oxalate, calcium or phosphate or of reduced phosphate reabsorption in the proximal tubule, nucleation of both calcium oxalate and calcium phosphate is expected to occur inside tubules around the bend in the Loop of Henle as was shown in models and in in vitro simulations [1, 12, 30]. The increase in calcium load that is required to cause this occurs with levels of hypercalcemia found in primary hyperparathyroidism. Blood levels of oxalate are less regulated than those of calcium and respond more to, for instance, dietary loads. The increase in oxalate load required to start nucleation is thus more common and already can occur after eating a bar of pure chocolate [31]. A likely cause of spontaneous calcium phosphate crystal formation in the loop of Henle is either an increase in plasma phosphate or a decrease in the fractional reabsorption of phosphate in the proximal tubule [12]. Due to the specific feature of constitutive high pH, especially in the longest loops of Henle [32], drugs of which the solubility is pH sensitive can also precipitate there [33]. After the loop of Henle the formation of aggregates becomes more likely as crystals emerging from different nephrons can meet each other [1]. In stone 1 the oldest central part is such an aggregate. The theoretical time frame for particle retention in the Ducts of Bellini, plugs, is tens of minutes [1, 12]. The observed timeframe lies within two-and-a-half hours. The time at which recurrent stone formers excreted 200 μ aggregates, in the plug size range after ingesting an oxalate load [34]. Aggregation will be enhanced when more crystals emerge from the nephrons, thus when supersaturation is increased and when the ability of the urine to inhibit crystal agglomeration is reduced. The latter for instance due to reduced citrate excretion [6, 7]. In daily life the combination of low agglomeration inhibition and low citrate excretion occurs as a result of a high-protein/high-salt diet [34]. This is exactly the type of “Western” diet that has been linked to increased risk for urolithiasis. Plug formation as described above was found in 50% of human kidney tissues obtained from patients with calcium oxalate nephrolithiasis who underwent nephrolithotomy or partial nephrectomy before the era of ESWL [18]. Calcium salt plugs were found within the tubular lumen, attached to the tubular walls or internalized into the tubular cells. In the study that linked stone formation to a reduced ability to prevent crystal agglomeration and reduced excretion of citrate, two of the patients were documented to form and excrete more than 50 stones per year [6]. This translates into a minimal growth time for a complete stone of around 1 week.

**Table 3:** Timeline of stone formation

Type of precipitate	Timeframe	Setting
Crystals in proximal tubule [15, 29]	Less than a minute	Extreme blood levels
Crystals in distal nephron parts [1, 12, 30]	Minutes	High blood levels
Plugs in duct of Bellini [16–18]	Up to 25 min high renal load, acidification problem	
Large aggregates in urine [35]	One-and-a-half hours	Oxalate load in stone formers
Fast stone formation [6]	1–2 weeks	Absent inhibition of agglomeration
Average stone formation	5 years	Lifestyle risk factors
Slow stone formation	23 years	Papillary plaques

Thus, plugs can be formed quickly. Since a plus remains exposed to urine, outgrowth is not limited by low supply of new material and stone formation may be faster. An encouraging finding is that plugs are more prevalent then stones [16–18]. Apparently there is some balance between plug formation and plug removal. A likely removal mechanism is flushing out (drinking), possibly demonstrated by the presence of dilated ducts. For pH sensitive material dissolution of a plug could theoretically play a role. Calcium oxalate stoneformers without clear hyperoxaluria or hypercalciuria have the lowest presence of tubular plugs. In these patients the plug formation was only linked to a reduced ability to prevent crystal growth. It must be noted that the procedure that was used to measure crystal growth inhibition does not separate effects on crystal growth from effects on crystal aggregation. Following the reasoning above, a majority of this risk-factor-free group will have formed stones through a fixed-particle (plaque) mechanism. They represent the end of the timescale where stone formation can take decades.

In summary we present the timeline of two stones that may represent the two mechanisms of stone formation.

One stone grew slowly but surely over two decades to be removed at a 3 cm size. Its origin is clouded in the past but does coincide with two events of renal trauma. At the time the stone revealed itself by causing colic pain the patient showed no risk factors for stone formation and was considered to be a true “idiopathic” stoneformer. This may well be the type of stoneformer for whom the existing lifestyle measures, which aim to reduce the driving force for crystallization, do not prevent stone formation efficiently, as the first step towards stone formation has already been taken. After the stone was removed three renal calcifications remained in place that can form the nidus for new stones. Whether these will again take decades to grow, what will initiate and speed up the growth process and what can be done to prevent or slow down that growth process are questions for which at present no answers exist. Answering these questions will require more insight into the process of stone growth.

The second stone is an example of a stone that was started from a situation of extreme renal overloading. It grew in a few years, and for this patient, clear risk factors can be identified in his lifestyle. Unfortunately, the extremely high BMI of this patient reflects a low compliance with the lifestyle changes that are needed to prevent new stone formation.

## Materials and methods

### CT scan

µCT scans were performed and reconstructed at the Applied Molecular Imaging Erasmus MC facility using the Quantum FX (PerkinElmer). The acquisition parameters for ex vivo scans were 90 kV, 160 µA with field of view of 20 mm and 360° rotation in 1 step. The acquisition time was 4.5 min.

### Determining stone growth by radiocarbon

Radiocarbon bomb-pulse dating was utilized to determine the ages of the stones layers and the core starting point following the method suggested in [36]. Using the CT scans the growth starting point and best dissection planes for each stone were determined. Stones were cut in half with a diamond disk saw of 0.3 mm thickness. Four powder samples were taken from each with 0.3 mm clean steel drill bit along the growth radius from the center to the outer surface, not going deeper in the material than 0.5 mm to avoid signal averaging over the significant period of time. Eight samples were processed to graphite following the procedure described in [36, 37]. Samples were analyzed on STAR AMS installation in ANSTO together with corresponding quality control material. Ages were determined by calibrating the radiocarbon measurements results with the radiocarbon calibration program CALIB 7.1.0 using the dataset of [38].

The other halves of the stones were used to do the elemental PIXE analyses with ion beam microprobe on ANTARES accelerator at ANSTO [39]. Measurements were done with the proton beam over the flat cut surface along the longest axis by scanning a strip of about 1.4 mm wide with ~20 µm resolution.
